# Comparison of Metabolic Syndrome Indicators in Two Samples of Central and South Americans Living in the Washington, D.C. Area in 1993–1994 and 2008–2009: Secular Changes in Metabolic Syndrome in Hispanics

**DOI:** 10.3390/ijerph14080881

**Published:** 2017-08-05

**Authors:** Regina Gill, Robert T. Jackson, Marguerite Duane, Allison Miner, Saira A. Khan

**Affiliations:** 1 Department of Nutrition and Food Science, University of Maryland, College Park, MD 20740, USA; reginam.gill@gmail.com (R.G.); bojack@umd.edu (R.T.J.); aminer@gmail.com (A.M.); 2 Department of Family Science, Georgetown University, Washington, DC 20007, USA; mduanemd@gmail.com

**Keywords:** central and South Americans adults, understudied groups, metabolic syndrome, obesity, heart disease, diabetes, epidemiology

## Abstract

The Central and South American populations are growing rapidly in the US; however, there is a paucity of information about their health status. *Objectives*: we estimated the prevalence of metabolic syndrome (MetS) and its individual components from two cohorts of Central and South Americans. *Methods*: This cross-sectional, medical record extraction survey sampled 1641 adults from a Washington, D.C clinic. A questionnaire was used to collect socio-demographic, medical history, anthropometric, biochemical, and clinical data. *Results*: among the 1993–1994 cohort, the MetS prevalence was 19.7%. The most prevalent MetS components were low high-density lipoprotein (HDL) cholesterol (40.4% men and 51.3% women), elevated triglycerides (40.9% men and 33.1% women), and high body mass index (BMI) ≥ 25 kg/m^2^ (27.6% men and 36.6% women). The overall prevalence of MetS in the 2008–2009 cohort was 28%. The most common abnormal metabolic indicator was an elevated BMI ≥ 25 kg/m^2^ (75.6%). 43.2% of men and 50.7% of women had HDL levels below normal, while the prevalence of hypertriglyceridemia was 46.5% and 32.5% for men and women, respectively. *Conclusion*: the prevalence of MetS was significantly greater in 2008–2009 compared with 1993–1994 (*p* ≤ 0.05). Dyslipidemia and high BMI have increased. Although similar components were identified in both the 1993–1994 and 2008–2009 study populations, the risks of MetS have increased over time.

## 1. Introduction

Metabolic syndrome (MetS) is a cluster of abnormalities that occur together in an individual and are associated with an increased risk of developing cardiovascular disease (CVD) and type 2 diabetes mellitus (DM) [1]. Abnormalities considered characteristic of the MetS include glucose intolerance, hyperinsulinemia, high plasma triglycerides, decreased high-density lipoprotein (HDL) cholesterol, hypertension, central obesity, pro-inflammatory states, and pro-thrombotic states, all of which increase the risk of developing CVD and DM [2]. MetS is closely associated with a generalized metabolic disorder called insulin resistance, in which tissue responsiveness to the normal action of insulin is impaired [3]. Being overweight or obese, physical inactivity, genetic factors, and poor diet are the underlying causes of the MetS [4]. 

Among United States (US) adults generally, the prevalence of MetS (as derived from the National Health and Nutrition Examination Survey (NHANES) III (1988–1994)) was estimated to be between 21.8% [5] and 23.9% [2], but seems to increase with age [5]. The prevalence of MetS among participants from the San Antonio Heart and Framingham Offspring Studies in the early to mid 1990s ranged from 21.3% (in white females) to 32.8% (in Mexican American females) using the National Cholesterol Education Program Adult Treatment Panel III (NCEP-ATP III) criteria [1]. In later studies, the prevalence of MetS was estimated to be 26.7% [6] and 34.5% [7] among subjects aged 20 years and older from NHANES 1999–2000. These studies suggest that the prevalence of MetS increased over time, especially as rates of obesity increased. 

Much of the information about chronic diseases in the US is derived from large national datasets such as the NHANES surveys, which are conducted to ascertain the current health and nutrition status of non-Hispanic whites, non-Hispanic blacks, and Hispanic Americans of primarily Mexican, Puerto Rican, and Cuban descent living in the US. Despite the plethora of information on these established Hispanic Americans, generalizations should not be made to other Hispanic subgroups, which have not been included in the large national surveys. According to Nath, “findings from one Hispanic subgroup cannot be applicable or extrapolative to other Hispanic subgroups because each subgroup’s social histories, cultural identities, health behaviors, and genetic compositions are unique” [8]. Thus, despite the growing body of available information on the commonly studied groups of Americans, very little is known about the prevalence of MetS among Central and South Americans, particularly those who are recent immigrants and whose numbers are increasing rapidly in the US.

There were two separate samples collected from different time periods (1993–1994 and 2008–2009). The objectives of this study were to examine how the prevalence of MetS has changed over this time period. We also sought to estimate the overall prevalence by gender and of individual risk components as well as the overall prevalence of MetS by ethnic group (country of origin) of Central and South Americans utilizing the Spanish Catholic Center. 

## 2. Materials and Methods

### 2.1. Metabolic Syndrome Assessment

Each subject was assessed for the presence of MetS. The MetS was defined using a modified harmonized definition that identified subjects if three or more of the following were present: (1) body mass index (BMI) ≥ 25 kg/ m^2^, (2) fasting plasma glucose ≥ 110 mg/dL, (3) HDL cholesterol < 40 mg/dL for men and < 50 mg/dL for women, (4) TGC ≥ 150 mg/dL, (5) blood pressure ≥ 130/85 mm Hg [3]. Data for waist circumference (WC) measurements were not available; as a result, a BMI ≥ 25 kg/ m^2^ was used for both men and women as an index to mean the subject was overweight. BMI was calculated as weight (measured to the nearest 0.1 kg) divided by the square of height (measured to the nearest 0.1 cm) and was categorized into four groups: underweight (<18.5 kg/m^2^), normal weight (18.5–24.9 kg/m^2^), overweight (25.0–29.9 kg/m^2^), and obese (>30.0 kg/m^2^), according to the World Health Organization (WHO) criteria [9].

### 2.2. Study Populations

Cross-sectional studies were conducted to assess the prevalence of MetS and its individual components in two separate samples of adult males and females attending the Spanish Catholic Center (SCC) medical clinic in Washington, D.C. One sample was collected in 1993–1994 and another mutually exclusive sample was collected during the 2008–2009 for group differences based on country of origin with a larger sample size using the same data collection methods and questionnaires. The SCC is a private, non-profit agency serving Latinos and the immigrant community. SCC has locations in Washington, D.C. and Montgomery County, Maryland, with a total of four clinics. SCC serves low-income populations who largely may not have health insurance. The SCC clinic used the federal guidelines for assessing the poverty level of their patients. Data for this study were obtained from the Washington, D.C. clinic, which is located in a heavily Hispanic neighborhood. Preventative and curative medical and dental services are provided based on income, but are usually free or at a modest cost. The countries represented in this data collection included (largest to smallest) El Salvador, Honduras, Peru, Guatemala, Bolivia, Argentina, Columbia, Nicaragua, Bolivia, Puerto Rico, Ecuador, Paraguay, Panama, Chile, Venezuela, and others. We reported group differences on the largest population in our database from El Salvador in the last table of the results. 

The 2008–2009 sample consisted of 1044 adults aged 18 years and older, who reported being from any Central or South American country, and who had fasting lipid profile measurements available. The 1993–1994 sample consisted of 607 adults, also aged 18 years and older, who also reported being from Central and South America [10]. On each occasion, medical records were extracted by a systematic sampling procedure, where the first subject was chosen at random using a statistical analysis system (SAS) (Version 9.2; SAS Institute Inc., Cary, NC, USA) random number generator; then, every third record was selected thereafter until the desired sample sizes were attained. The desired sample size was calculated based on a 3% margin of error, 95% confidence and 50% expected MetS prevalence. We increased the sample size for the second sampling period in order to attain group differences. The target sample sizes for 1993–1994 and 2008–2009 were 600 and 1014, respectively. Protocols for both time periods were approved by the human subjects committees of the SCC and by the Institutional Review Board at the University of Maryland, College Park, code # 09-0264 approved on 20 April 2009. 

Both the 1993–1994 and 2008–2009 surveys had the same research questions and the data collection sheets had questionnaires and variables that were similarly structured. The biochemical indices were analyzed by the same commercial laboratory.

The questionnaire was used to obtain sociodemographic information reported in patient files. Sociodemographic variables included age, gender, country of origin, state of residence, education, employment status, income, and years living in the US. Lifestyle variables included current smoking status and alcohol consumption. Medical history information was also collected to assess general health and to determine if subjects had been told by a physician that they had diabetes, hypertension, or heart problems.

Blood was taken by a trained nurse after an overnight fast. Biochemical indices were analyzed by Quest Diagnostics Incorporated. The concentrations of fasting plasma lipids and glucose were measured using standardized blood analysis techniques. Blood glucose was analyzed using an automated enzymatic method [11]. Cholesterol and high density lipoprotein (HDL) cholesterol were both analyzed enzymatically. Triglyceride (TGC) levels were analyzed by automated spectrophotometry [12]. Low density lipoprotein (LDL) cholesterol was mathematically derived using the Friedewald formula for subjects with TGC levels of 400 mg/dL or less [13].

### 2.3. Statistical Analysis

Data are displayed as means ± standard deviations (SD) for continuous variables, and as frequencies and percentages for categorical variables. From the 2008–2009 sample, underweight subjects (with a BMI < 18.5 kg/m^2^ (*n* = 5)) were excluded, as well as those subjects receiving insulin therapy for treatment of diabetes (*n* = 2). From the 607 SCC 1993–1994 study, we excluded eight subjects aged <18 years. Differences between the mean values of MetS components based on year of attendance at the medical clinic and country of origin were assessed by analysis of variance (ANOVA), with a Tukey–Kramer post-hoc test to accommodate groups with unequal sample sizes. A chi square test was used to compare the prevalence rates of MetS and MetS components in the two populations and analyses stratified by gender. A Student’s t test compared independent means between study populations, as well as between men and women, for selected variables. All tests were two-tailed. A significance level was set at *p* ≤ 0.05, and analyses were performed using the statistical analysis system (SAS) (Version 9.2; SAS Institute Inc., Cary, NC, USA).

## 3. Results

A total of 1,641 men and women were selected for comparison. Ages ranged from 18 to 87 years. The final 1993–1994 and 2008–2009 sample sizes were 599 and 1,042, respectively. Anthropometric, biochemical, and clinical characteristics of the two study populations stratified by gender are shown in Table 1. 

### 3.1. The 1993–1994 Cohort 

Among the 1993–1994 subjects, the mean and SD for age was 37 ± 14 years, respectively, and 66.1% were women (*n* = 396). Women were significantly older (*p* ≤ 0.05) and had significantly higher body mass index (BMI) ≥ 25 kg/m^2^ and high-density lipoprotein(HDL) cholesterol < 40 mg/dL for men and < 50 mg/dL for women levels than men (*p* ≤ 0.05). Men had higher fasting blood glucose levels (not significant) and triglycerides (TGC) ≥ 150 mg/dL levels (*p* ≤ 0.05) than women. 

### 3.2. The 2008–2009 Cohort 

Among the 2008–2009 subjects, the mean and SD for age was 42 ± 13 years, respectively, and 67.6% were women (*n* = 704). These women had higher BMI (*p* ≤ 0.05) and HDL levels (*p* ≤ 0.05) than men. Men had higher mean levels of TGC (*p* ≤ 0.05), fasting glucose (*p* ≤ 0.05), LDL cholesterol (*p* ≤ 0.05), and lower mean levels of HDL cholesterol (*p* ≤ 0.05) than women. 

### 3.3. Comparison of Two Cohorts

Men and women in the 2008–2009 sample were older than the men and women in the 1993–1994 sample. In each time period, however, the women were slightly older than the men. The mean weights and heights of men and women in the 2008–2009 cohort was greater than those of the 1993–1994 cohort. The BMIs of men and women in the 2008–2009 survey were greater than the BMIs of women and men in the earlier 1993–1994 survey; however, only the BMIs of men were statistically greater (*p* ≤ 0.05). TGC levels of men and women were similar over the two study periods (*p* ≥ 0.05), but in each period men had higher levels than women.

Metabolic risks were more common among men compared with women; for example, Table 1 shows that the mean TGC value in men, as well as the mean LDL cholesterol concentration in men and women, exceeded the harmonized defined optimal cutoff criteria. The mean HDL cholesterol value for women in 1993–1994 was also lower than the harmonized cutoff criterion and lower than the mean for women from 2008–2009. Overall, when the two populations were compared as a whole, the 2008–2009 subjects had significantly higher BMI, lower fasting glucose, and lower systolic and diastolic blood pressures (*p* ≤ 0.05).

When comparing men from the 1993–1994 and the 2008–2009 cohorts, many of their characteristics were similar. Men from the 1993–1994 study had significantly higher systolic and diastolic blood pressures (*p* ≤ 0.05), whereas men from the 2008–2009 study were significantly heavier, had a higher BMI, and were older (*p* ≤ 0.05). When the women from each study were compared, a number of differences were identified between them. Besides being significantly heavier, taller, and older (*p* ≤ 0.05), the women from the 2008–2009 study had lower mean metabolic risk values; some were significantly lower, such as fasting glucose, total cholesterol, and blood pressure. 

Country of origin was self-reported by the subjects. Nearly 50% of each study cohort reported being from El Salvador. The majority of subjects (63%) from the 1993–1994 cohort resided in Washington, D.C., whereas the majority of subjects (57%) from the 2008–2009 cohort resided in Maryland and were employed with a median income of approximately $7800 to $13,000 per year, respectively. Subjects were either married (40% in 1993 vs. 45% in 2008–2009) or single (46% in 1993–1994 vs. 43% in 2008–2009). 

In the 2008–2009 cohort, the average number of dependents was 2.6 and 2.3 for men and women, respectively. A total of 69% of subjects had primary or secondary education, while only 23% spoke English fluently. Only about 100 (11%) subjects had medical insurance. The average number of years that subjects had been living in the US was 8.8 years. In the 1993–1994 study, by contrast, nearly all (96%) subjects obtained primary or secondary education, only 2% of subjects had medical insurance, and only 8% spoke fluent English.

Table 2 shows the overall prevalence of the MetS and individual MetS components among male and female Central and South American subjects from the 1993–1994 and 2008–2009 study periods. The prevalence of MetS was significantly greater (*p* ≤ 0.05) for subjects in 2008–2009 compared with subjects in 1993–1994. The prevalence of low HDL levels was very high among all subjects in both study periods.

The most prevalent MetS indicators among men in 1993–1994 were low HDL (40.4%) and high TGC (40.9%), while low HDL (51.3%) and high BMI (36.6%) were most prevalent for women. Women had a significantly higher (*p* ≤ 0.05) prevalence of low HDL compared to men in both study samples (51.3% vs. 40.4% in 1993–1994 and 50.7% vs. 43.2% in 2008–2009). 

For the men and women in the 2008–2009 cohort, the most common abnormal indicators of the MetS was being overweight, i.e., BMI ≥ 25 kg/ m^2^ , (78.4% of men and 74.3% of women), as defined by the NCEP ATP III cut-offs [14], low HDL, and high TGC. The overall prevalence of hypertension was 9.6% among men and women in 2008–2009. The difference in the prevalence rates of hypertension between the two cohorts was statistically significant (*p* ≤ 0.05) for both men (20.2% vs. 11.2%) and women (18.7% vs. 8.8%). 

The prevalence of MetS component clustering among men and women from the 1993–1994 study is presented in Table 3. Clustering of abnormal MetS components was more prevalent among men compared to women for most components. Overall, 152 men had one or more MetS components, with the largest percentage having only one component (31.5%). However, women were more likely than men to have only one component (32.3%). Men had a higher prevalence of clustering of three components, indicative of the MetS, compared to women (14.8% vs. 13.4%). Based on the number of subjects with three, four, or five MetS components, the prevalence of MetS for men was 19.7% (*n* = 40) and the prevalence for women was also 19.7% (*n* = 78). 

In the 2008–2009 sample, 29.3% of men had one abnormal component, 26.0% of men had two abnormal components, 26.9%, 5.9%, and 1.2% of men had three, four, or all five abnormal components of MetS. When compared to men, these percentages were generally lower for women, with values of 29.7, 19.7, 19.9, 4.7, and 0.4%, respectively. The total prevalence of MetS in this study population was 27.9%. The prevalence for men was 34.0% (*n* = 115) and the prevalence for women was 25% (*n* = 176). 

In both the 1993–1994 and 2008–2009 samples, the most frequently reported country of origin was El Salvador. There were a total of 804 Salvadorian subjects in the study; 296 were from the 1993–1994 study and 508 were from the 2008–2009 study. The difference in MetS risks over time was determined in this subset of the study subjects. Table 4 shows the anthropometric and metabolic characteristics of subjects from El Salvador from the 1993–1994 and 2008–2009 studies.

The mean BMI for subjects from 1993–1994 was 28.1 ± 12.5, while subjects from 2008–2009 had a mean BMI of 29.3 ± 5.2; this difference is statistically significant (*p* ≤ 0.05). The percentages of subjects who were overweight and obese in 1993–1994 were 30.6% and 31.6%, respectively. The percentages of subjects who were overweight and obese in 2008–2009 were 22.4% and 34.6%, respectively. An elevated BMI of ≥ 25 kg/m^2^ was more prevalent among the 2008–2009 study subjects. There was a statistically significant (χ^2^, *p* ≤ 0.05) difference between the prevalence of elevated BMI in the two samples. This statistical difference was seen at a BMI of ≥ 25 kg/m^2^ and ≥ 30 kg/m^2^. 

Many of the biochemical variables for El Salvadorians were similar in the two cohorts. However, the mean diastolic blood pressure (DBP) of the 2008–2009 subjects was significantly lower (72.0 ± 12.1 vs. 80.2 ± 11.1) compared to the 1993–1994 Salvadoran subjects (*p* ≤ 0.05). This was the only statistically significant difference between the MetS indicators between the two cohorts. Most of the means of the biochemical indicators were higher in the 1993–1994 sample, although not significantly so. 

Prevalence of hypertriglyceridemia, elevated SBP, and high total cholesterol were higher among subjects from 2008–2009; however, these differences were not statistically significant (*p* > 0.05). 

## 4. Discussion

Metabolic syndrome increases the risk of cardiovascular disease and DM (diabetes mellitus) in individuals. Ours is one of the first studies to show that the prevalence of MetS (metabolic syndrome) has increased over the last 15 years in low-income, immigrant Central and South Americans to the US. In 1993–1994, the overall prevalence of MetS was 19.7%, while in 2008–2009 the prevalence was 29.7%, and it was higher in males (34.0%) than in females (25%) in those later years (Table 3). Our findings are consistent with those of Ford et al. [6], who also showed an increase in MetS prevalence over time as between NHANES III (1988–1994) (23.1%) and subsequent iterations of NHANES 1999–2000 (26.7%). Ford noted that the increased prevalence of MetS was mainly associated with an increase in the percentage of subjects with an increased waist circumference and hypertriglyceridemia. Our study (Table 2) shows a similar increase in the prevalence of MetS with a concomitant increase in the prevalence of overweight and obesity measured by BMI. Other studies [15] have discussed the changing definitions of MetS over time; while obesity has been a focus over time, both WC and BMI have been widely used [16,17]. Research has noted the comparability of the two measures [18] and the latest definition called for Central and South Americans to use values similar to that of South Asians until more data on this population has been revealed.

In a later study of MetS prevalence and risk factors based on the NHANES 2003–2006 survey data, Ervin found that the overall MetS prevalence was 34%. That prevalence was higher than that found from data taken from the NHANES 1999–2000, but also higher than both of our overall estimates in 1993–1994 (19.7%) and in 2008–2009 (29.7%). 

Our findings in Central and South Americans were similar to those found in other Hispanic American populations [19,20]. For example, in a population-based study in Peru, the PREVENCION study, the most common metabolic abnormality in women was low HDL (high-density lipoprotein) cholesterol (60.9%); whereas, in men it was hypertriglyceridemia (52.0%) followed by low HDL cholesterol (32.5%). Similar to our findings, abnormal fasting plasma glucose was the least common component of MetS for men (5.4%) and women (5.0%) [20]. Additionally, in studies conducted in various Central and South American countries, Espinosa–Larranaga et al. concluded that approximately 50% of the populations in Argentina, Chile, Paraguay, Peru, and Colombia are overweight and more than 15% are obese. They also found that, in Chile, 39.3% of the population had low HDL (male <40 mg/dL, female <50 mg/dL) cholesterol; and, in Venezuela, men had higher levels of triglycerides (47%) with low levels of HDL cholesterol (40%) than women [18]; these results are consistent with our findings.

Mexican-Americans from San Antonio, Texas have also been identified [1] as having prevalent dyslipidemia, with hypertriglyceridemia (48.9% men and 36.8% women), low HDL cholesterol (53.6% men and 60.4% women), and large WC (waist circumference, 31.2% men and 56.4% women), but Mexican Americans also had a much higher prevalence of hypertension (44.1% men and 36.9% women) than our Central and South American subjects. Cheal et al. found that “being overweight, in combination with high plasma triglycerides and/or low HDL cholesterol, was a powerful predictor of having the metabolic syndrome [21].” 

In our study, dyslipidemia (elevated plasma triglycerides and low HDL cholesterol levels) were very common. This pattern of dyslipidemia was also seen in Venezuelan subjects with MetS [22]. Martínez–Ortiz, et al., found a positive association between the dietary patterns of Latin Americans and included a staple-based dietary pattern characterized by an increased intake of refined grains (white bread and rice), added sugar, coffee, legumes, red meat, and an increased use of palm oil for cooking, with low HDL cholesterol leading to an increased risk of myocardial infarction in Costa Rican adults [23]. 

There were several gender-related differences between our two cohorts. The prevalence of MetS increased substantially between 1993–1994 and 2008–2009, particularly in men. The most significant encouraging changes over time in metabolic profiles were seen in women. For example, systolic and diastolic blood pressures, TGC, and fasting plasma glucose values were significantly lower for women in the 2008–2009 sample compared to 1993–1994, indicating that certain risks decreased. Men, however, did not show the same decreased prevalence in MetS indicators. DBP (diastolic blood pressure) was the only variable which was significantly greater in the 1993–1994 population (Table 2). 

Mean weights, BMIs, and ages were greater in our 2008–2009 study participants. These results are similar to that found by others showing that MetS prevalence increases with increases in BMI [3,24,25,26] and age [20,27].

In the 1993–1994 cohort of Central and South Americans, the most prevalent metabolic abnormalities were low HDL cholesterol, hypertriglyceridemia, and elevated BMI (Table 2). The prevalence of having a BMI ≥ 25 kg/m^2^ and low HDL was significantly greater in women compared to men. 

The 2008–2009 cohort of Central and South Americans experienced a very similar pattern of metabolic abnormalities. The most prevalent abnormalities in this latter cohort were elevated BMI, hypertriglyceridemia, and low HDL cholesterol (Table 2). The most important contributor to the increased MetS prevalence between 1993 and 2008 were the significant increases in the number of subjects meeting and exceeding the BMI criterion of ≥ 25 kg/m^2^ (*p* ≤ 0.05). 

As can be seen from Table 3, the overall prevalence of MetS for men and women is similar, but the clustering of abnormal indicators varies between the sexes. The percentages of subjects with one or more MetS components are not consistently greater in one sex or the other. Women have higher prevalence estimates of one and four abnormal components compared to men, respectively, who have higher prevalence estimates of two, three, and five components (Table 3). The prevalence estimates for clustering of MetS components found in the 2008–2009 study population (shown in text) were similar, in that women had a higher prevalence of one abnormal component compared to men, but men had higher prevalence estimates for two through five components, indicating that not only has the prevalence of MetS increased dramatically among men, but also that certain components (low HDL, high TG, and high BMI) have increased more dramatically in men than in women. 

Contrary to our findings, Medina–Lezama et al. found that Peruvian women had higher prevalence estimates for one through five components of the MetS [20]. In their study, 31.8% of women had MetS with 3, 4, or all 5 components as opposed to 17.8% of men. However, consistent with our findings, Cheal et al. found that the men had higher prevalence estimates of 1, 2, 4, and all 5 components of the MetS [21]. 

We performed an analysis looking at the differences in MetS risk factors between subjects that originated from the largest Hispanic sub-group in our study. In both the 1993–1994 and 2008–2009 studies, El Salvador was the most frequently reported county of origin. As shown in Table 4, few significant mean differences in MetS characteristics exist between the previous and present cohort of Salvadorians. Differences that do exist follow the same pattern as that seen when the two study samples were compared, which are an increase in the prevalence of high BMI and a decrease in the prevalence of hypertension among subjects from 2008–2009. The results from the anthropometric characteristics, biochemical variables, and MetS characteristics of Salvadorian subjects are consistent with the other Central and South American subjects used in our study as a whole, thus indicating similar metabolic risks. However, in an examination comparing El Salvador to other countries individually, differences in metabolic risks may exist.

Strengths of this study include its focus on an understudied segment of the Hispanic population that is rapidly growing in the US. Secondly, our study provides evidence for a secular increase in MetS in a group that may lack adequate resources to obtain adequate medical and heath interventions. MetS prevalence is valuable for increasing the awareness and knowledge of this problem in this understudied population. A limitation may be that users of the medical clinic may not be representative of the Hispanic population living in the Washington, D.C. Metropolitan Area. Another limitation may be the lack of WC data available for collection, since the newer harmonized MetS definition focus on central obesity. Our study may underestimate the prevalence of MetS by substituting BMI for WC. We also do not have a representative sample, and may by biased because this was a convenience sample. There may also be acculturation differences in the groups in the US compared to their host countries.

## 5. Conclusions

The prevalence of MetS (metabolic syndrome) has increased over time. When the two study populations were compared, it was determined that the prevalence of MetS was higher in 2008–2009 subjects, with a more noticeable increase among men. Subjects from 1993–1994 and 2008–2009 had the same clustering of metabolic abnormalities. Our study, as well as other studies of Central and/or South Americans, suggests that being overweight, in combination with high plasma triglycerides and/or low HDL (high-density lipoprotein) cholesterol, is a potent predictor of the MetS in these groups. For these subjects with dyslipidemia and elevated BMI (high body mass index), medical treatment, drug therapy, and dietary changes could help lower the risk of MetS and subsequent CVD (cardiovascular disease) and DM (diabetes mellitus). 

## Figures and Tables

**Table 1:** Characteristics of the Central and South American study population by gender in 1993–1994 and 2008–2009 ^1^.

Description	1993–1994				2008–2009			
	*n*	Men	*n*	Women	*n*	Men	*n*	Women
Age (years)	203	35.1 ± 19.4	396	38.9 ± 14.2	338	41.5 ± 13.6 ^3^	704	42.8 ± 13.2 ^2^
≤ 34 (%)	119	58.6	180	45.5	114	33.7	237	33.6
35-44 (%)	40	19.7	95	24.0	96	28.4	166	23.6
45-54 (%)	23	11.3	59	14.9	66	19.5	173	24.6
55-64 (%)	14	6.9	40	10.1	46	13.6	90	12.8
65 (%)	7	3.5	22	5.6	16	4.7	38	5.4
Weight (kg)	163	71.2 ± 11.4	350	64.6 ± 12.8	331	77.3 ± 12.5 ^3^	701	69.3 ± 14.2 ^2^
Height (cm)	91	164.8 ± 8.2	198	153.0 ± 7.3	323	165.4 ± 7.4	688	154.7 ± 6.9 ^2^
BMI (kg/m^2^)	91	26.1 ± 3.8	198	28.4 ± 5.2	323	28.3 ± 4.1 ^3^	688	29.0 ± 5.6
SBP (mmHg)	185	123.3 ± 19.4	369	119.8 ± 21.2	336	119.7 ± 17.7 ^3^	703	115.7 ± 18.5 ^2^
DBP (mmHg)	185	82.2 ± 11.1	369	79.2 ± 11.2	336	72.9 ± 12.1 ^3^	703	71.0 ± 10.9 ^2^
TC (mg/dL)	203	192.7 ± 44.7	389	195.7 ± 48.5	332	191.5 ± 38.9	694	190.5 ± 36.7 ^2^
HDL-c (mg/dL)	201	43.8 ± 11.6	387	49.9 ± 12.4	332	42.9 ± 10.4	694	50.5 ± 12.7
LDL-c (mg/dL)	192	116.5 ± 34.6	379	116.9 ± 31.8	320	116.7 ± 33.5	684	113.3 ± 31.3
FPG (mg/dL)	202	102.0 ± 41.9	389	99.2 ± 46.2	329	99.9 ± 31.3	686	93.3 ± 27.9 ^2^
TGC (mg/dL)	203	167.6 ± 132.3	389	136.5 ± 83.2	331	163.8 ± 97.9	691	136.4 ± 78.6

^1^ Data are mean ± S.D, unless otherwise specified; ^2^ Significantly different from women in the 1993–1994 population; ^3^ Significantly different from men in the 1993–1994 population. BMI: body mass index; SBP: systolic blood pressure; DBP: diastolic blood pressure; TC: total cholesterol; HDL: high-density lipoprotein cholesterol; LDL: low-density lipoprotein cholesterol; FPG: fasting plasma glucose; TGC: triglycerides.

**Table 2:** Prevalence of individual metabolic syndrome components and the metabolic syndrome among Central and South American men and women from 1993–1994 and 2008–2009.

**Component**	**No. and % with Abnormal MetS Components**			
	**1993–1994 (*n* = 599)**		**2008–2009 (*n* = 1042)**	
	**Men (*n* = 203)**		**Men (*n* = 338)**	
	*n*	%	*n*	%
High BMI *	56	27.6	265	78.4 ^1^
Low HDL ^†^	82	40.4	146	43.2
Hypertension	41	20.2	38	11.2 ^1^
High FPG	31	15.3	42	12.4
High TGC	83	40.9	157	46.5
MetS prevalence	40	19.7	115	34.0 ^1^
**Component**	**Women (*n* = 396)**		**Women (*n* = 704)**	
	***n***	**%**	***n***	**%**
High BMI	145	36.6 ^2^	523	74.3 ^1^
Low HDL ^†^	203	51.3 ^2^	357	50.7 ^2^
Hypertension	74	18.7	62	8.8 ^1^
High FPG	44	11.1	59	8.4 ^2^
High TGC	131	33.1 ^2^	229	32.5 ^2^
MetS prevalence	78	19.7	176	25.0 ^1,2^

Data are %. * BMI ≥ 25 kg/m^2^; HDL: high-density lipoprotein cholesterol; FPG: fasting plasma glucose; low HDL ^†^: male <40 mg/dL, female <50 mg/dL; TGC: triglycerides; ^1^ significantly different from 1993–1994 population; ^2^ significantly different from men.

**Table 3:** Prevalence of metabolic syndrome clustering among Central and South American men and women from 1993–1994 and 2008–2009.

**MetS Components**	**Men (*n* = 203)**	**Men (*n* = 338)**
No. of components	1993–1994	2008–2009
1	31.5 *	25.9 *
2	23.6	19.9
3	14.8	19.2
4	3.4	4.7
5	1.5	2.4
Total No. of subjects w/Metabolic Syndrome	40	115
Prevalence of Metabolic Syndrome	19.7	34
**MetS Components**	**Women (*n* = 396)**	**Women (*n* = 704)**
No. of components	1993–1994	2008–2009
1	32.3 *	27.7 *
2	19.9	17.6
3	13.4	13.9
4	5.8	6.1
5	0.5	1.0
Total No. of subjects w/Metabolic Syndrome	78	176
Prevalence of Metabolic Syndrome	19.7	25

* Values are percentages.

**Table 4:** Anthropometric and metabolic characteristics of subjects from El Salvador from 1993–1994 and 2008–2009 ^1^.

Component	1993–1994	2008–2009
*n*	296	508
**Anthropometric characteristics**		
Weight (kg)	68.3 ± 12.5	73.2 ± 14.2 ^2^
Height (cm)	157.6 ± 9.6	157.7 ± 7.8
BMI (kg/m^2^)	28.1 ± 4.8	29.3 ± 5.2 ^2^
**Biochemical variables**		
Fasting Plasma Glucose (mg/dL)	99.0 ± 35.6	96.6 ± 32.0
Triglyceride (mg/dL)	153.5 ± 115.1	150.9 ± 87.4
Systolic Blood Pressure (mmHg)	120.0 ± 19.0	118.2 ± 19.1
Diastolic Blood Pressure (mmHg)	80.2 ± 11.1	72.0 ±12.1 ^2^
High Density Lipoprotein (mg/dL)	45.7 ± 11.6	46.2 ± 11.4
Low Density Lipoprotein (mg/dL)	112.6 ± 30.8	114.4 ± 30.5
Total Cholesterol (mg/dL)	188.5 ± 38.8	190.3 ± 37.0
**Metabolic syndrome characteristics**		
High BMI (% population)	34.5	79.5 ^2^
High FPG (% population)	12.8	9.8
High TGC (% population)	34.8	38.0
Hypertension (% population)	18.2	11.2 ^2^
Low HDL (% population) ^3^	65.9	65.6

^1^ Means ± SD, unless otherwise specified; ^2^ significantly different from the 1993–1994 population, *p* ≤ 0.05 (Student’s *t* test for continuous variables and chi-square test for categorical variables); ^3^ HDL < 50 mg/dL for the entire population.
